# What is preventing psychotherapy case studies from having a greater impact on evidence-based practice, and how to address the challenges?

**DOI:** 10.3389/fpsyt.2022.1101090

**Published:** 2023-01-12

**Authors:** Jochem Willemsen

**Affiliations:** Louvain Psychotherapy Research Group, Psychological Sciences Research Institute, Université catholique de Louvain, Louvain-la-Neuve, Belgium

**Keywords:** case study, psychotherapy, evidence-based practice, training, methodology

## 1. Introduction

Case studies are an essential part of evidence-based practice (EBP) because they provide a type of evidence for clinical practice that cannot be provided by other types of research. Case studies demonstrate how (not just which) change processes operate to achieve positive outcome, and how these processes can be influenced within the context of an individual treatment. Even in cases with positive outcome there are many barriers and resistances that can be documented in a case study and that are very helpful for practitioners to learn about. Case studies are also very well suited to demonstrate individualized, qualitative, and non-symptomatic changes through psychotherapy (1). In this sense, case studies contribute to the emergence of the personalized approach to treatment outcome. Through the focus on a single patient, case studies contain insights and knowledge in a format and at a level that is coherent with the practitioner's way of thinking. Clinical reasoning and case conceptualization are always about “thinking in cases” (2, 3). The key element here is particularization, not generalization: while efficacy studies demonstrate whether interventions work on average in a group, case studies demonstrate how they work with individual patients through a detailed description of therapeutic microprocesses. This is lost if case study approaches are not built into the fabric of EBP. However, there are several obstacles that are currently preventing case study research from having a greater impact on EBP.

## 2. The persuasiveness issue

Although case-based research complements other types of research in unique ways, it is often considered inferior to sample-based research. Efficacy studies apply a very straightforward and simple causal logic which consists of checking whether the same cause (under sufficiently similar conditions) produces the same effect. If a cause and an effect regularly follow each other (for instance in a sample of patients) it can be deduced that the cause (for instance a psychological intervention) creates an effect (for instance symptom reduction). Moreover, statistical significance testing provides efficacy studies with a clear binary criterion for determining the evidential value of psychological interventions. For these reasons, Cartwright (4) calls this type of studies “clinchers”: if the assumptions are met, the conclusion can be deduced with certainty. This makes them very persuasive for researchers, policy makers and the general public. At the same time, efficacy studies have limited value for clinical practice and cannot be the sole source of EBP (4). As pointed out in the introduction, other types of research evidence, including case studies, should be considered in the context of EBP. However, these other types of evidence have a less straightforward causal logic and have less clear criteria for determining the evidential value of interventions. In case study research, causality is situated in a complex psychological process that takes place at the level of the unique case, and case studies (at best) provide evidence that makes a case for a conclusion. Case studies never allow to deduce with certainty a clear yes/no answer to the question what works. Cartwright calls this type of research “vouchers”: in contrast to “clinchers,” they can only speak for a conclusion (4). This makes them less persuasive.

The persuasiveness issue cannot be solved by trying to emulate “clinchers.” What can improve their credibility is increasing the visibility of the strengths and impact of case study research, and increasing the awareness about new developments in the field. At the methodological level, specific approaches to rigorous data collection and data analysis for case studies have been developed (5, 6); quality criteria to improve the evidential value of clinical case studies have been proposed (7, 8). Moreover, methods to generalize by comparing and aggregating case studies are available and used (9, 10). Others have argued that the use of data from psychotherapy trials for conducting individual-focused case studies can “improve the yield” of painstakingly collected data, but also increase the impact of research on practice (11). The difference between sample-selection and case-selection has been highlighted as a crucial parameter in the process of generalization (12). At the epistemological level, Stake (13) introduced the concept of naturalistic generalization to describe knowledge that is gained through personal or vicarious experiences, for instance by reading case studies. Moreover, the concept of statistical generalization, which is so important for EBP based on efficacy studies, must be complemented with the concept of analytic generalization (14) which underpins the use of case studies for theory building (15). In the past, it has often been suggested that case studies are only useful for generating descriptions of and hypothesis about phenomena that are not well understood (“context of discovery”). However, these recent methodological and epistemological developments have shown that case studies can also contribute to the “context of justification.” That is, under the right conditions, they can provide rigorous tests of theory and therapeutic technique (16, 17).

## 3. The lack of framework issue

Within the community of case study “producers” and case study “users” (researchers, trainers, trainees, students, practitioners, policy makers), there is a lack of a shared framework of criteria to assess the quality of case studies. This problem can be situated at different levels. At the level of ethics, there are inconsistencies in the policies used by psychotherapy training institutions, universities and publishers that oversee the ethical aspects of case study research. Ethical committees within academic or clinical institutions often struggle to evaluate project applications that involve case study research, either because they don't see its scientific merit (see Section 2), or because their experience with the evaluation of sample-based studies is unhelpful when it comes to assessing the ethical intricacies of a case study project. At the level of the methodology, there are no tools to evaluate the quality of case studies. Existing frameworks and tools for evaluating the quality of research evidence (e.g., GRADE) categorize case studies as “low-quality evidence.” This is because these tools apply the criteria for efficacy studies to case reports. As a consequence, policy-makers undervalue the importance of case studies in the development of EBP. Organizations that promote and support EBP, such as the Joanna Briggs Institute and the Critical Appraisal Skills Programme, have developed checklists for rating the quality of qualitative research and case reports. However, these tools are developed for medical research, and they are diagnosis-oriented. Moreover, these tools and checklist operate on the basis of a categorical difference between qualitative and quantitative research, whereas case studies often take the form of mixed-method research.

The lack of framework issue can be addressed by developing ethics, methodology, and epistemology consensual frameworks for case study research. The general criteria for good science (objectivity and generalization) need to be adapted to case study research, in which the focus is on the individual and in which context plays a central role. In the field of social sciences, much work has been done on developing the methodological and epistemological principles that underpin case study research (14). This literature needs to be explored for relevant concepts and frameworks. Truijens et al. (18) argue that a precondition for EBP is the development of a theory of evidence that is clear on what should be evidenced to be useful and valid for psychological interventions. An important step toward the development of a framework is Kaluzeviciute's (19) Case Study Evaluation-tool (CaSE), the first tool that offers a framework and a checklist to evaluate the evidential value of case studies in the field of psychotherapy. This tool needs to be completed, disseminated, and implemented more broadly.

## 4. The accessibility issue

Case studies remain difficult to access by researchers, trainers, practitioners, and policy-makers. For case studies to have an impact on EBP, they should be easily accessible through searchable digital databases. However, current databases that are most often used for the development of EBP like Science Citation Index, PubMed and PsycInfo, do not allow to search the field of case study research efficiently. Case studies are often difficult to find with regular search terms, which makes it difficult to find relevant (sets of) case studies. Practitioners who want to look up case studies that can inform their work are not able to systematically identify relevant case studies. As a result, experiences and insights from clinical practice are insufficiently transformed into knowledge that can be shared and taught and, in a sense, each practitioner must discover the richness of clinical work anew. Practitioners write case studies in the context of their training and for professional development, but almost none of this work is available to other practitioners or for researchers (20). Training institutions have archives of case studies in the form of doctoral theses that are not cataloged and classified, and therefore not accessible.

The accessibility issue can be addressed by increasing the visibility of existing case study research and by making case studies more easily accessible. Practitioners need to be able to access case study research *via* different platforms and in different formats, for instance journals articles, online database of vignettes… A coordinated effort is needed to map available case study resources, for instance in the archives of training institutions. Several initiatives have been taken in the past. The Ulm Textbank (21) and the Psychoanalytic Research Consortium (22) are collections of recorded and transcribed therapy sessions that provide a rich resource for case study research. Miller (in 2004) and Iwakabe (in 2005) laid the foundation for building a searchable database of case studies. Their initiatives inspired a network of researchers from Ghent University, Université catholique de Louvain, and the University of Essex to create the Single Case Archive (www.singlecasearchive.com), an online searchable database of +3,400 published case studies that can be used by researchers, trainers, practitioners, and students. In the context of the Single Case Archive project, the first comprehensive review of case studies was published (23).

## 5. Discussion

The research-practice gap in the field of psychotherapy has been described as a lack of integration between the findings disseminated by researchers and the decisions made in the consulting room by therapists (19). This gap has negative consequences for the application of research findings in mental health services as well as on the development of research—supported psychological treatments. Case studies are an important means to reduce this gap because they provide templates of how to integrate basic research and knowledge into applied work at the individual case level. Reducing the research-practice gap should not be a matter of promoting the systematic uptake of research findings and other evidence-based practices into routine practice (top-down implementation). Rather, therapists should be engaged as learners that learn from their own and others' experiences and that learn from sharing and reflective on these experiences. The flexible case study research approach is a means to stimulate, support, and improve these learning processes, while considering different learning styles. For that reason, the above-mentioned obstacles need to be addressed urgently.

## Author contributions

The author confirms being the sole contributor of this work and has approved it for publication.
